# Supplementary figures and images for: An LTR retrotransposon in the promoter of a PsMYB10.2 gene associated with the regulation of fruit flesh color in Japanese plum
Source: Hortic Res. 2022 Sep 13;9:uhac206. doi: 10.1093/hr/uhac206 (PMC9715577; doi:10.1093/hr/uhac206)

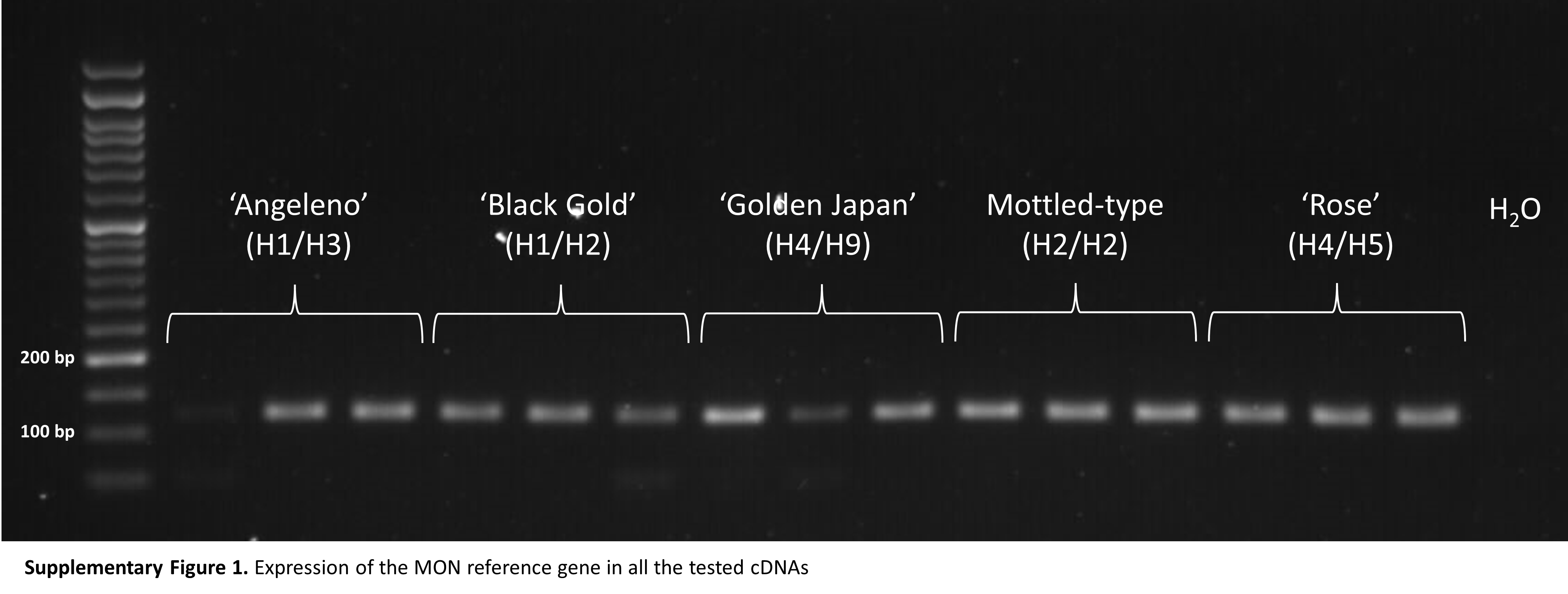

Supplement: Web_Material_uhac206 [file web_material_uhac206.zip › Supplementary Figure 1. cDNA internal control.tif]

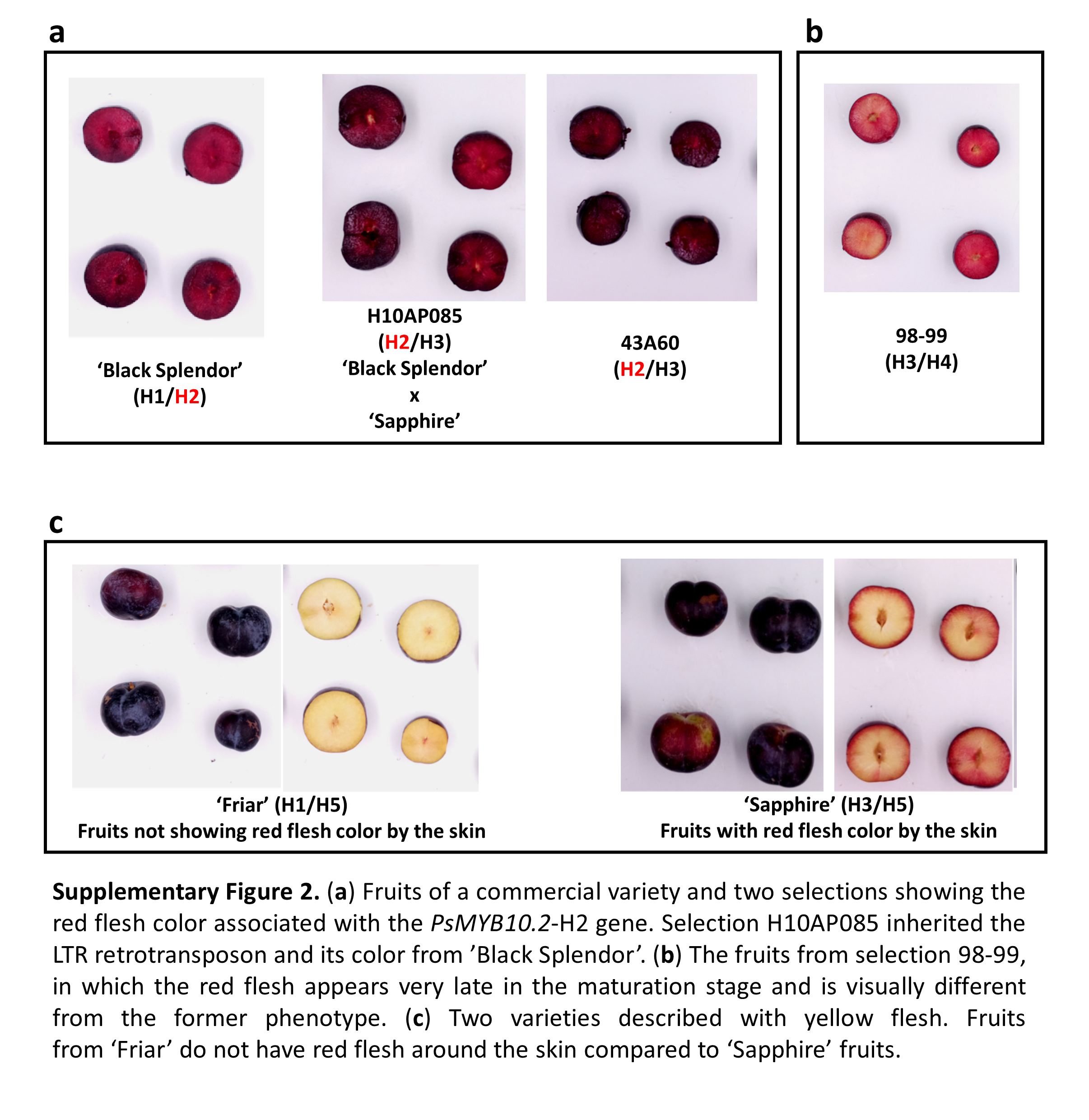

Supplement: Web_Material_uhac206 [file web_material_uhac206.zip › Supplementary Figure 2. Fruits with different coloration.tif]
